# Gross Motor Function Disorders in Patients with Alternating Hemiplegia of Childhood

**DOI:** 10.34763/jmotherandchild.2020241.1935.000003

**Published:** 2020-07-29

**Authors:** Agnieszka Stępień, Katarzyna Maślanko, Maciej Krawczyk, Witold Rekowski, Anna Kostera-Pruszczyk

**Affiliations:** 1 Department of Rehabilitation, Józef Piłsudski University of Physical Education, Warsaw, Poland; 2 Center of Functional Rehabilitation Orthos, Warsaw, Poland; 3 Psychosocial Foundation of Health and Rehabilitation, Department of Rehabilitation, Józef Piłsudski University of Physical Education, Warsaw, Poland; 4 Department of Neurology, Medical University of Warsaw, Warsaw, Poland

**Keywords:** alternating hemiplegia of childhood, rare diseases, gross motor function disorders, child development, physiotherapy

## Abstract

**Background:**

Alternating hemiplegia of Childhood (AHC) is a rare disease manifested by transient episodes of hemiplegia and other neurological disorders. Delayed motor development has been reported in patients with AHC, but detailed features of the motor impairment have not been described so far.

**Aim:**

The aim of the study was to evaluate gross motor function between attacks in a group of Polish patients with AHC.

**Materials and methods:**

The interictal gross motor function was assessed using the Gross Motor Function AHC scale, which consisted of 41 motor tasks. The study group consisted of 10 patients with AHC older than 2 years of age. The control group consisted of 30 age- and gender-matched subjects. The results achieved in each of the 41 tasks by the study subjects were compared to the results obtained with controls using the non-parametric Mann–Whitney U-test. In tasks 38–41, mean times were compared between the study subjects and controls.

**Results:**

The study revealed gross motor function impairment in patients with AHC. The greatest differences compared to controls concerned such skills as standing on toes, walking on toes, walking on heels, as well as running and hopping on one leg and on alternate legs. Significant impairment of the motor function of the upper limbs was also found.

**Conclusions:**

The study confirmed motor function impairment between attacks in patients with AHC. The study findings may indicate the need to introduce individualised physiotherapy management of patients with AHC.

## Introduction

Alternating hemiplegia of childhood (AHC) is a rare neurological disease characterised by recurrent hemiplegic attacks and other paroxysmal symptoms, abolished with sleep. The incidence is estimated at 1 per 1,000,000 births (1). AHC was first described by Verret and Steele in 1971 (2). Most cases of AHC are attributed to de novo mutations in the *ATP1A3* gene coding for alpha-3 catalytic subunit of the Na+/K(+)-ATPase (3,4,5).

Paroxysmal symptoms include episodes of hemiplegia, bilateral hemiplegia, quadriplegia, dystonia, tonic spells, seizures and abnormal eye movements (5,6,7,8). Also observed are respiratory difficulty, dysarthria, dysphagia, facial dyskinesia or athetosis, as well as autonomic disturbances such as bradycardia, stridor, bronchospasm, changes in skin color, excessive constriction or dilation of the pupils, vomiting, diarrhoea, sweating, hyperthermia, hypothermia or excessive salivation (5,6,7,8,9,10,11). The hemiplegic episodes vary in frequency and duration (6). The clinical status of AHC sufferers varies during and between attacks (5). Intellectual and motor development is impaired in most patients with AHC (10,12). Tone abnormalities, choreoathetosis, ataxia and motor clumsiness are observed (8). Fine motor skills, cognitive development and behaviour are also affected (5).

## Aim of the Study

The aim of the study was to assess gross motor function between attacks in a group of Polish patients with diagnosed AHC and to describe the disease-specific motor deficits between attacks. The results of the study may allow the design of a dedicated physiotherapy programme for patients with AHC.

## Materials and Methods

The study was conducted from November 2014 until the end of May 2015. Contact with the patients was facilitated by the Polish Association for Persons with AHC (AHC-PL), which is a nongovernmental, not-for-profit and support organisation promoting the interests of and acting on behalf of patients with AHC.

The subjects were included in the study when AHC was diagnosed by a neurologist or geneticist based on the Aicardi criteria (13). To qualify for a detailed assessment of gross motor capabilities, the subject had to be >2 years of age. The exclusion criteria included a hemiplegic attack during the study or feeling unwell, which made administration of the tests impossible.

A control group was established for comparison with gross motor function assessments in AHC patients. For each study subject, three age (+ 6 months)- and gender-matched controls were randomly selected, i.e. a total of 30 controls. The exclusion criteria for controls included the following: participation in competitive sports; post-injury status or surgical operation within the past 12 months; or feeling unwell, which made completing the study impossible.

The parents of the children and the adults participating in the study were informed about its aims, and they signed the consent to participate in the study and publish data form. The study was approved by the Senate Research Ethics Committee at the Józef Piłsudski University of Physical Education in Warsaw, Poland (SKE 01–07/2015).

Fourteen patients with AHC, aged 1.2–30.1 years, and/or their carers expressed their wish to participate in the study. All of the subjects were members of the Polish Association for persons with AHC (AHC-PL). Two children below 2 years of age were not included in the detailed assessment of gross motor function because of their young age, and two patients were excluded from the study due to an ongoing hemiplegic attack. Ultimately, the study was completed by 10 subjects (four females and six males) >2 years of age. Mutations of *ATP1A3* were identified in seven subjects. The information on age, height and weight of the study patients, as well as that of the age- and gender-matched controls, is presented in **Table 1**.

**Table 1:** Characteristics of the study (AHC) and control groups

**Study group (AHC)**						**Control group**				

**No.**	**Gender**	**Height (cm)**	**Weight (kg)**	**Age (years)**	**Mutation of ATP1A3**	**No.**	**Gender**	**Mean height (cm)**	**Mean weight (kg)**	**Mean age (years)**
1	F	83	11.5	2.5	+	1.1; 1.2; 1.3	F	89.8	13.6	2.5
2	M	104	14	3.5	+	2.1; 2.2; 2.3	M	100.3	15	3.5
3	M	104	19	3.5		3.1; 3.2; 3.3	M	103	19	3.5
4	F	124	23	6.2	+	4.1; 4.2; 4.3	F	121.3	22	6.6
5	F	128	22	9.1		5.1; 5.2. 5.3	F	141	31	8.9
6	M	132	26	9.1	+	6.1;6.2; 6.3	M	142.7	37	9.2
7	M	164	60	12.8		7.1; 7.2;7.3	M	159.7	53	12.9
8	M	188	50	20.3	+	8.1; 8.2; 8.3	M	181	74	20.5
9	F	154	52	24.3	+	9.1; 9.2; 9.3	F	169	62.3	24.3
10	M	184	70	30.1	+	10.1;10.2;10.3	M	182.5	82.5	30.3

The study consisted of two parts. The subjects with diagnosed AHC and/or their carers first completed a questionnaire providing personal details and history of the disease, and next, the gross motor function was assessed.

AHC patients and/or their carers reported typical signs, such as plegic attacks, tonic/dystonic attacks, nystagmus, deviation of the eyes, episodic respiratory difficulties and episodic autonomic disturbances. The plegic attacks occurred from a few to 20–30 times a month and lasted from a few minutes to several days. A detailed list of signs and symptoms, frequency and duration of the attacks in individual study subjects is presented in **Table 2**.

**Table 2:** Characteristics of the study (AHC) group – signs

**No.**	**Unilateral Attacks**	**Bilateral attacks**	**Signs**	**Frequency (number of attacks per month)**	**Average duration of episode**	**Medicines**
1	+	+	Plegic attacks Tonic/dystonic attacks Nystagmus Deviation of the eyes Episodic respiratory difficulties Episodic autonomic disturbances	1–2	30 min–2 h	Yes
2	+	+	Plegic attacks Tonic/dystonic attacks Nystagmus Deviation of the eyes Episodic respiratory difficulties Episodic autonomic disturbances	4–8	7 days–several days	No
3	+	+	Plegic attacks Tonic/dystonic attacks Ataxia	A few	A few minutes to few hours	Yes
4	+	+	Plegic attacks Tonic/dystonic attacks Nystagmus Deviation of the eyes	1–30	A few minutes to 12 h	No
5	+		Tonic/dystonic attacks Nystagmus Episodic autonomic disturbances	5–30	30 s–1 min	Yes
6	+	+	Plegic attacks Tonic/dystonic attacks Deviation of the eyes Episodic respiratory difficulties	10	A few hours–2 days	Yes
7	+	+	Plegic attacks Tonic/dystonic attacks Nystagmus	1–20	A few minutes to a few hours	Yes
8	+	+	Plegic attacks Tonic/dystonic attacks Deviation of the eyes Episodic respiratory difficulties Episodic autonomic disturbances	One to a few	A few hours to a few days	No
9	+	+	Plegic attacks Tonic/dystonic attacks Nystagmus Episodic respiratory difficulties Plegic attacks	17–20	A few minutes to a few days	No
10	+	+	Tonic/dystonic attacks Deviation of the eyes Episodic respiratory difficulties	Few – 20	Several minutes to a few hours	Yes

Gross motor function was assessed using the Gross Motor Function AHC (GMF AHC) scale proposed by the authors, comprising 41 motor tasks ranked by order of appearance of motor skills in human motor development (**Table S1**). The GMF AHC scale was based on various published motor function scales, mainly on the Hammersmith Functional Motor Scale – Expanded (HFMSE) and the Gross Motor Function Measure (GMFM), and it included other motor tasks proposed by the authors of the study and which were selected after appropriate pilot studies. The GMF AHC scale used 23 items from the HFMSE and 2 items from the GMFM. Because of the differences in age and motor function status among the patients, after an initial assessment of some of them, 16 new motor tasks were added.

The HFMS was originally developed to assess function in non-ambulant children with spinal muscular atrophy. The scale was later modified, extended (HFMSE) and validated (14,15,16). Authors used the following sub-tests from HFMSE: 1. Lifts head from supine position; 2. Moves from lying to sitting position; 3. Duration of sitting; 4. Duration of long sitting; 5. Places right hand on head while sitting, hand touching the head above the level of ears; 6. Places left hand on head while sitting, hand touching the head above the level of ears; 7. Places two hands on head while sitting, hands touching the head above the level of ears; 8. Moves from sitting to lying position; 9. Moves from supine position to lying on side; 10. Rolls over from supine position to prone position on the right; 11. Rolls over from prone position to supine position on the right; 12. Rolls over from supine position to prone position on the left; 13. Rolls over from prone position to supine position on the left; 14. Lifts head from the prone position; 15. Props on forearms; 16. Props on extended arms; 17. Performs four-point kneeling; 18. Performs crawling; 20. Executes supported standing; 21. Stands unsupported; 22. Squats; 33. Ascends stairs without rail; 34. Descends stairs without rail.

The GMFM was originally designed to assess gross motor function in children with cerebral palsy (17). It has also been used and validated in children with other diseases associated with significant motor impairment (18,19,20). The authors used the following sub-tests from GMFM: 24. Walks forward; 28. Runs.

The new motor tasks added by the authors were as follows: 19. Gets to standing position from a supine position; 23. Gets off a chair without using arms; 25. Stands on toes; 26. Walks on toes; 27. Walks on heels; 29. Hops on both legs; 30. Hops on right foot; 31. Hops on left foot; 32. Hops on alternate legs; 35. Catches a ball with both hands, stands – ball 25 cm, five attempts; 36. Throws a tennis ball – dominant hand, stands, five attempts; 37. Kicks ball – dominant leg, stands, ball 20 cm, 3 m, five attempts; 38. Stands on right leg with eyes open, hands crossed on chest; 39. Stands on left leg with eyes open, hands crossed on chest; 40. Stands on right leg with eyes closed, hands crossed on chest; 41. Stands on left leg with eyes closed, hands crossed on chest.

The validation of the GMF AHC prior to the study was impossible to perform due to the extremely small number of patients. The patients and controls were tested by four qualified physiotherapists. The tests were carried out on patients in the lying, sitting and standing positions. Tasks 1–37 could score 0, 1 or 2 points (0= task not performed; 1= task partially performed; 2= task normally performed), while in tasks 38–41, the time it took the subject to perform the task was measured. Tasks 38–41 had to be performed in triplicate up to 15 seconds in duration, and the best result was recorded. Instructions for each motor task were given to the subject verbally, followed by a demonstration by the investigator.

The results for the study subjects were analysed using the SPSS software. The results achieved in each of the 41 tasks by the study subjects were compared to the results for controls using the non-parametric Mann–Whitney *U*-test. In tasks 38–41, the mean times were compared between the study subjects and controls. The results were considered statistically significant at *p*<0.05.

## Results

Tests for gross motor function in subjects aged 2–30 years found statistically significant differences between the study group and controls in 22 tasks (Table 3). The study subjects achieved significantly worse results in the following sub-tests: 19. Gets to standing from a supine position; 21. Stands unsupported; 22. Squats; 23. Gets off a chair without using arms; 24. Walks forward; 25. Stands on toes; 26. Walks on toes; 27. Walks on heels; 28. Runs; 29. Hops on both legs; 30. Hops on right leg; 31. Hops on left leg; 32. Hops on alternate legs; 33. Ascends stairs without rail; 34. Descends stairs without rail; 35. Catches a ball with both hands; 36. Throws a tennis ball; 37. Kicks ball – dominant leg; 38. Stands on right leg with eyes open; 39. Stands on left leg with eyes open; 40. Stands on right leg with eyes closed; 41. Stands on left leg with eyes closed.

**Table 3:** Gross Motor Function AHC scale – differences between study (AHC) and control groups

**Item**	**Gross Motor Function AHC Study (AHC) group**		**Gross Motor Function AHC Control group**		**p**

	**Mean ± SD**	**SE**	**Mean ± SD**	**SE**	
1. Lifts head from supine position	1.90 ± 0.32	0.10	1.93 ± 0.25	0.05	0.73
2. Lying to sitting position	1.90 ± 0.32	0.10	2.00 ± 0.00	0.00	0.08
3. Sitting position	2.00 ± 0.00	0.00	2.00 ± 0.00	0.00	1.00
4. Long sitting position	2.00 ± 0.00	0.00	2.00 ± 0.00	0.00	1.00
5. Right hand to head in sitting position	1.90 ± 0.32	0.10	2.00 ± 0.00	0.00	0.08
6. Left hand to head in sitting position	1.90 ± 0.32	0.10	2.00 ± 0.00	0.00	0.08
7. Two hands to head in sitting position	1.90 ± 0.32	0.10	2.00 ± 0.00	0.00	0.08
8. Sitting to lying position	2.00 ± 0.00	0.00	2.00 ± 0.00	0.00	1.00
9. Supine position to lying on side	2.00 ± 0.00	0.00	2.00 ± 0.00	0.00	1.00
10. Rolls from supine position to prone position over right	1.90 ± 0.32	0.10	2.00 ± 0.00	0.00	0.08
11. Rolls from prone position to supine position over right	1.90 ± 0.32	0.10	2.00 ± 0.00	0.00	0.08
12. Rolls from supine position to prone position over left	1.90 ± 0.32	0.10	2.00 ± 0.00	0.00	0.08
13. Rolls from prone position to supine position over left	1.90 ± 0.32	0.10	2.00 ± 0.00	0.00	0.08
14. Lifts head from prone position	2.00 ± 0.00	0.00	2.00 ± 0.00	0.00	1.00
15. Props on forearms	2.00 ± 0.00	0.00	2.00 ± 0.00	0.00	1.00
16. Props on extended arms	2.00 ± 0.00	0.00	2.00 ± 0.00	0.00	1.00
17. Achieves four-point kneeling	1.90 ± 0.32	0.10	2.00 ± 0.00	0.00	0.08
18. Crawls	2.00 ± 0.00	0.00	2.00 ± 0.00	0.00	1.00
19. Gets to standing from a supine position	1.60 ± 0.84	0.27	2.00 ± 0.00	0.00	0.01*
20. Executes supported standing	1.80 ± 0.63	0.20	2.00 ± 0.00	0.00	0.08
21. Stands unsupported	1.60 ± 0.84	0.27	2.00 ± 0.00	0.00	0.01*
22. Squats	0.96 ± 0.61	0.19	1.79 ± 0.46	0.08	0.00*
23. Gets off a chair without using arms	1.60 ± 0.84	0.27	2.00 ± 0.00	0.00	0.01*
24. Walks forward	1.60 ± 0.84	0.27	2.00 ± 0.00	0.00	0.01*
25. Stands on toes	0.50 ± 0.53	0.17	1.90 ± 0.31	0.06	0.00*
26. Walks on toes	1.18 ± 0.90	0.28	2.00 ± 0.00	0.00	0.00*
27. Walks on heels	0.70 ± 0.82	0.26	2.00 ± 0.00	0.00	0.00*
28. Runs	0.94 ± 0.88	0.28	1.94 ± 0.13	0.02	0.00*
29. Hops on both legs	1.30 ± 0.82	0.26	2.00 ± 0.00	0.00	0.00*
30. Hops on right foot	0.40 ± 0.84	0.27	1.90 ± 0.31	0.06	0.00*
31. Hops on left foot	0.40 ± 0.84	0.27	1.83 ± 0.38	0.07	0.00*
32. Hops on alternate legs	0.43 ± 0.56	0.18	1.56 ± 0.48	0.09	0.00*
33. Ascends stairs without rail	1.25 ± 0.78	0.25	1.92 ± 0.20	0.04	0.00*
34. Descends stairs without rail	1.25 ± 0.78	0.25	1.92 ± 0.20	0.04	0.00*
35. Catches a ball with both hands	1.20 ± 0.79	0.25	1.93 ± 0.25	0.05	0.00*
36. Throws a tennis ball	1.60 ± 0.84	0.27	2.00 ± 0.00	0.00	0.01*
37. Kicks ball – dominant leg	1.60 ± 0.84	0.27	2.00 ± 0.00	0.00	0.01*
38. Stands on right leg with eyes open	3.58 ± 5.01	1.59	11.92 ± 4.59	0.84	0.00*
39. Stands on left leg with eyes open	3.78 ± 5.07	1.60	11.81 ± 4.43	0.81	0.00*
40. Stands on right leg with eyes closed	2.22 ± 3.52	1.11	8.82 ± 5.97	1.09	0.00*
41. Stands on left leg with eyes closed	1.93 ± 2.48	0.79	9.03 ± 6.03	1.10	0.00*

SD, standard deviation; SE, standard error.

*p* calculated using the Mann–Whitney *U*–test;

* *p*<0.05.

## Discussion

Studies of AHC are relatively recent and, to date, cases of patients from a number of countries have been reported (10, 12, 21,22,23,24,25,26). The aim of the present study was to provide detailed characteristics of gross motor dysfunction in AHC patients. To the best of our knowledge, to date, no other such study has been performed.

There are no available tools to assess gross motor function in AHC or to compare gross motor function in subjects who vary greatly in age. The motor tasks included in the Gross Motor Function AHC scale developed for the purpose of the present study allow the assessment of motor function in particular body parts and, as a result, a detailed description of the motor dysfunction in each subject. The Gross Motor Function AHC is based on items of the HFMSE and the GMFM, to which new sub-tests have been added. The advantage of the GMF AHC is its ability to assess the subject's performance in different positions, from lying prone and supine to standing, during both static and dynamic activities. However, the scoring system does not assess the quality and precision of the movements, which should be addressed in further studies.

We found gross motor impairment in AHC subjects, in agreement with the reports from other authors, who observed delayed development in most individuals with AHC (5,10).

In our study, AHC subjects achieved the worst results in the following tasks: standing and walking on toes, walking on heels, running, hopping on one leg and hopping on alternate legs. Walking abnormalities in AHC were also noted in other published studies (5). Kirshenbaum et al. (27) studied mice with mutations in the gene responsible for AHC. The animals had less-stable gait, walked with shorter steps and had poorer postural stability than wild-type mice (27).

In the present study, two subjects aged 2.5 and 3.5 years were not able to walk independently.

The results of the present study mostly indicate impaired control of the muscles producing plantar and dorsal flexion of the foot, stabilising muscles of the foot, as well as muscles straightening the knee and hip joints, which are active during hopping, walking and running. Additionally, the test results suggest impaired coordination and fluidity of movement, with a narrow base of support. Tests performed in a lying position did not reveal any dysfunction of trunk muscles. However, significantly impaired function of the upper limbs was observed during catching and throwing. Such a pattern of muscle function abnormalities is consistent with disturbances in consecutive phases of development and indicates generally delayed development. Interestingly, inability to perform alternate movements of the trunk and extremities was observed in all study patients. Our findings allow the development of AHC-specific physiotherapy management. They cannot be compared to other studies because, to our knowledge, there have been no published studies of this kind. It would be worthwhile to conduct similar studies involving a larger group of patients from different countries.

Undoubtedly, the limitation of this research is the large discrepancy in age of the study subjects. Taking it into consideration, the authors randomly selected three age- and gender-matched controls for each AHC subject. This method of qualification was intended to reduce the effect of age on the results.

The results of our research expand our knowledge about major motor disorders in AHC patients. It can be assumed that a dedicated physiotherapy management might improve the condition of AHC patients, but this requires confirmation in further studies.

## Conclusion

Gross motor impairment is observed in patients with AHC between attacks. The present study mostly indicates impaired control of the muscles responsible for the functioning of the lower extremities. The study findings may indicate the need to introduce individualised physiotherapy management of patients with AHC, tailored to their motor development, but this requires further research.

## Figures and Tables

**Table S1:** Gross Motor Function AHC

**Item No.**	**Item**	**Points: 0**	**Points: 1**	**Points: 2**
1	Lifts head from supine position†	Unable	Head is lifted, but through side flexion or with no neck flexion. Held for a count of 3	In supine position, head must be lifted in midline. Chin moves towards chest. Held for a count of 3
2	Lying to sitting position†	Unable	Turns into prone position or towards floor	Able to do by lying on side
3	Sitting†	2 hands for support	1 hand for support, for a count of 3	No hand support, for a count of 3
4	Long sitting †	2 hands for support	1 hand for support, for a count of 3	No hand support, for a count of 3
5	Right hand to head in sitting position† Hand touches head above level of ears	Unable	Can only bring right hand to head by flexing head	Able to bring right hand to head. Head and trunk remain stable
6	Left hand to head in sitting position† Hand touches head above level of ears	Unable	Can only bring left hand to head by flexing head	Able to bring right hand to head. Head and trunk remain stable
7	Two hands to head in sitting position† Hands touch head above level of ears	Unable	Able to place hands on head but only using head flexion or side tilt	Able to place both hands on head, arms free from side. Head and trunk remain stable
8	Sitting to lying position†	Unable or falls over	Able to lie down by flopping forwards and rolling sideways	Able to lie down in a controlled fashion through lying on side or using clothes
9	Supine position to side-lying position†	Unable	Can ½ roll only one way: either right or left	Able to ½ roll from supine position both ways
10	Rolls from supine position to prone position over right †	Unable	Turns to prone position by pulling/pushing on arms	Turns to prone position with free arms to the right
11	Rolls from prone position to supine position over right †	Unable	Turns to supine position using arms to push/pull with	Turns to supine position with free arms to the right
12	Rolls from supine position to prone position over left†	Unable	Turns to prone position by pulling/pushing on arms	Turns to prone position with free arms to the left
13	Rolls from prone position to supine position over left†	Unable	Turns to supine position using arms to push/pull with	Turns to supine position with free arms to the left
14	Lifts head from prone position†	Unable	Lifts head with arms in a forward position for a count of 3	Able to lift head up in prone position, arms by side for a count of 3
15	Props on forearms†	Unable	Holds position when placed for a count of 3	Able to achieve prop on elbows with head up for a count of 3
16	Props on extended arms†	Unable	Holds position when placed	Achieves hands support in prone position
17	Four-point kneeling †	Unable	Holds position when placed for a count of 3	Achieves four-point kneeling – head up for a count of 3
18	Crawling†	Unable	Moves all four points only once	Able to crawl forwards – moves all four points twice or more
19	Gets to standing position from a supine position§	Unable	Uses furniture	Achieves standing without furniture
20	Supported standing†	Can stand with hand support but needs knee/hip support in addition for a count of 3, unable	Able to stand with minimal trunk support (not hip) for a count of 3	Can stand using one hand support for a count of 3
21	Stands unsupported†	Stands only momentarily, unable	Stands independently for a count of 3	Can stand independently for more than a count of 3
22	Squats†	Unable	Initiates squat (>10%), uses arm support, or static <3 seconds	Squats with arms free (at least 90° of hip and knee flexion), static > 3 seconds
23	Gets off a chair without using arms§	Unable	Uses arms	Gets off independently without using arms
24	Walks forward‡	Unable	Walks <10 steps	Walks >10 steps
25	Stands on toes§	Unable	Stands <5 seconds	Stands >5 seconds
26	Walks on toes§	Unable	Walks with assistance, <3 m	Walks independently, >3 m
27	Walks on heels§	Unable	Walks with assistance, <3 m	Walks independently, >3 m
28	Runs‡	Unable	Runs <4.5 m	Runs >4.5 m, stops and returns
29	Hops on both legs§	Unable	<5 consecutive hops	>5 consecutive hops
30	Hops on right foot§	Unable	<5 consecutive hops	>5 consecutive hops
31	Hops on left foot§	Unable	<5 consecutive hops	>5 consecutive hops
32	Hops on alternate legs§	Unable	Does not hop on alternate legs and arms, <10 consecutive hops	Hops on alternate legs and arms, >10 consecutive hops
33	Ascends stairs without rail†	Unable	Ascends <4 stairs, arms free, any pattern or with arm support	Ascends 4 stairs, arms free, alternating feet
34	Descends stairs without rail†	Unable	Descends <4 stairs, arms free, any pattern or with arm support	Descends 4 stairs, arms free, alternating feet
35	Catches a ball with both hands, stands, ball 25 cm, 5 attempts§	Unable	Catches a ball with both hands, throws to a distance >1.5 m	Catches a ball with both hands >4 times
36	Throws a tennis ball, stands, dominant hand, 5 attempts§	Unable	Throws distance <1.5 m or throws to a distance >1.5 m < 4 times	Throws to a distance > 1.5 m, 4 or 5 times
37	Kicks ball – dominant leg, stands, ball 20 cm, 3 m, 5 attempts §	Unable	Kicks 3 m< 4 times	Kicks 3 m> 4 times
38	Stands on right leg with eyes open, hands crossed on chest §	Timer		
39	Stands on left leg with eyes open, hands crossed on chest §	Timer		
40	Stands on right leg with eyes closed, hands crossed on chest §	Timer		
41	Stands on left leg with eyes closed, hands crossed on chest §	Timer		

† Tasks based on the Hammersmith Functional Motor Scale Expanded (HFMSE);

‡ the Gross Motor Function Measure (GMFM) and

§ other motor tasks.
